# Correlation between early clinical exposure environment, attitudes toward basic medicine, and medical students’ basic science learning performance

**DOI:** 10.1186/s12909-019-1612-0

**Published:** 2019-06-03

**Authors:** Kung-Pei Tang, Chien-Yu Chen, Ming-Shun Wu, Tzu-Tao Chen, Bei-Wen Wu, Po-Fang Tsai

**Affiliations:** 1 Department of Education and Humanities in Medicine, School of Medicine, College of Medicine, Taipei Medical University, No.250, Wu-Hsing Street, Taipei, 11031 Taiwan; 2 Department of Anesthesiology, School of Medicine, College of Medicine, Taipei Medical University, No.250, Wu-Hsing Street, Taipei, 11031 Taiwan; 3 Department of Anesthesiology, Taipei Medical University Hospital, No.250, Wu-Hsing Street, Taipei, 11031 Taiwan; 4 Graduate Institute of Humanities in Medicine, College of Humanities and Social Sciences, Taipei Medical University, No.250, Wu-Hsing Street, Taipei, 11031 Taiwan; 5 Department of Internal Medicine, School of Medicine, College of Medicine, Taipei Medical University, No.250, Wu-Hsing Street, Taipei, 11031 Taiwan; 6 Division of Gastroenterology, Department of Internal Medicine, Wan Fang Hospital, Taipei Medical University, No.111, Sec. 3, Xinglong Rd., Taipei, 11696 Taiwan; 7 Department of Medical Education, Taipei Medical University Shuang Ho Hospital, No.291, Zhongzheng Rd., Zhonghe District, New Taipei City, 23561 Taiwan; 8 Division of Pulmonary Medicine, Department of Internal Medicine, Taipei Medical University Shuang Ho Hospital, No.291, Zhongzheng Rd., Zhonghe District, New Taipei City, 23561 Taiwan

**Keywords:** Early clinical exposure, Mentorship, On doctoring, Early student-patient contact, Preceptorship

## Abstract

**Background:**

Early clinical exposure (ECE) is viewed as a way to provide contexts of basic science and highlight its relevance to medical practice. However, very few studies have specifically looked into how the ECE experience contributes to students’ academic performance. The purpose of this study was to investigate whether ECE experiences (external cause) or students’ learning attitudes (internal cause) more closely correlated with medical students’ academic performance.

**Methods:**

Subjects who participated in the study comprised 109 s-year students at Taipei Medical University. Fifty of the 109 study participants were enrolled in an elective ECE program. The dependent variable in this study was the test score of a systems-based basic sciences (SBBS) course. Independent variables of the study included students’ attitudes and test anxiety towards the SBBS course, engagement/length of time spent in ECE, and the ECE learning environment. Data of students’ engagement in ECE, levels of their motivational beliefs and test anxiety, differences in the ECE learning environment, and the SBBS final test scores of these 109 respondents were collected for hierarchical multiple regression (HMR) analyses.

**Results:**

Results of the HMR analyses revealed that students’ test anxiety towards basic science and also the learning environment of the ECE had significant positive predictive power on their SBBS test scores.

**Conculsion:**

This study discovers that medical students’ academic performance in basic science correlates not only with their anxiety to testing, but even more so with the clinical environment they are exposed to. Hence we suggest including further investigations about different learning environments on ECE experiences in future studies.

**Electronic supplementary material:**

The online version of this article (10.1186/s12909-019-1612-0) contains supplementary material, which is available to authorized users.

## Background

Several current studies have described the early clinical exposure (ECE) experience and its positive contributions to medical education, but very few quantitative studies have further attempted to explain factors that contribute to a successful ECE experience. The purpose of this study is to investigate the correlations between medical students’ academic performance and personal or environmental factors. In regards to environmental factors, the authors examined how the exposure to ECE impacts students’ achievement, which includes an analysis of the hospital environment and the duration of the program. To determine the correlations with personal factors, the study has considered the roles of students’ self-efficacy, intrinsic values, and test anxiety and how each has influenced students’ academic performance.

### ECE as an asset to medical education

ECE (i.e., early student-patient contact or preceptorship) as a hegemonic educational model has been adopted by many medical schools throughout the world to close the gap between basic and clinical science; the model was derived from the Flexner Report [1, 2]. ECE is often defined as authentic human contact in a social or clinical context during the preclinical medical years [2–4]. In terms of the preclinical medical years, ECE should occur before the official clerkship and internship training programs [5].

ECE is viewed as the beginning of the process of professional socialization and the development of mentoring relationships, and is also seen as a way to provide contexts for basic science and its relevance to medical practice [6–10]. Many studies investigated outcomes of ECE and revealed that ECE programs motivate medical students in numerous ways [11–13]. For instance, it was concluded that the ECE experience provides positive motivation toward medical education and in turn improves students’ performance in examinations [3, 8, 14–16].

Başak, Yaphe [5] conducted a survey across Europe and found that observation, small group teaching, clinical bedside teaching, supervision and feedback, reflective journal writing, self-learning, case-based learning, lectures, and shadowing were common teaching and learning activities in ECE programs [5]. Ottenheijm, Zwietering [4] added that every fruitful ECE program should follow three educational principles: 1. maintain students’ learning cycle based on Kolb’s experiential learning; 2. emphasize the active role of students; and 3. provide timely supervision and feedback.

Başak, Yaphe [5] also indicated that ECE training mostly takes place in primary care settings, general practice clinics, department outpatient clinics, and hospital wards, with just a few programs taking place in the community. Besides experiential learning, medical students also engage in situational learning during the ECE preceptorship, while the community surrounding the clinic continues to shape the activity as a whole [17]. The learning process during ECE is therefore social and collaborative, so that outcomes of ECE experiences should be affected by the environment.

The ECE program at Taipei Medical University (TMU) is an elective 4-year course that recruits undergraduates from freshmen to seniors. The application process takes place every spring semester. This program screens applicants according to the priority of their chosen elective courses. All ECE program participants are grouped according to simple random sampling and assigned to three urban tertiary teaching hospitals. In each hospital, a group of attending physicians serve as mentors to the students. The mentor-mentee ratio is 1:1~1:2.

Students who participate in ECE programs have the following mandatory assignments: 1. 24 clinical trainee hours; 2. biweekly reflective journals during the semester; and 3. a one-time summary report of clinical observations. Activities during the clinical trainee hours are agreed upon between the mentor and mentee(s). Instead of a conventional course syllabus, a learning contract technique is applied to identify individual learning objectives for the clinical hours. However, students engage in typical ECE activities, such as shadowing, supervision and feedback, small group teaching, and self-directed learning. In addition to mandated assignments, students are invited to social gatherings organized by their mentors every 2 weeks.

Compared to other ECE programs, the ECE program at TMU has the following features: to encourage students’ self-directed learning, the TMU format is elective and has no rigid syllabus. Each participant determines his/her ECE learning objectives with his/her mentor. To maintain the continuity-based mentor-mentee relationship, this ECE format requires that each participant stay in the same hospital groups with one set of faculty members until he/she withdraws.

Considering that performance emerges through interactions between participants and the environment [17], we outlined the following differences among the three ECE groups in Table 1.

**Table 1:** Information of early clinical exposure (ECE) groups

Information	Group A	Group B	Group C
Distance from the university campus to the ECE hospital (in km)	0.27	5.2	9.3
Number of mentors	13	12	16
Number of mentees	17	17	16
Mentor’s specialty categories	12	10	11
Number of chief physicians as mentors	11	3	12
Student retention rate of each group	7/17 (41.1%)	10/17 (58.8%)	12/16 (75%)
Proportion of students who fulfilled the 24 clinical trainee hours and the biweekly reflective journals during the semester	7/17 (41.1%)	12/17 (70%)	12/16 (75%)

After each semester, students have the option to stay in or opt out of the ECE course. In group A, 41.1% of mentees remained enrolled in the spring semester 2016, with 58.8% in group B and 75% in group C (Table 1). Although ECE course participants are required to take 24 trainee hours per semester, not every student met this requirement. Attendance records of fall 2016 showed that group C had the highest fulfillment rate of 75%, followed by group B of 70%, and then group A of 41.1%. This is an interesting finding because students in group C had the longest commute between their university campus and the ECE hospital (90–120 min back and forth via public transportation), followed by group B students of 60 min, and with the least travel time for group A students of 10-min walking time.

In this study, we aim to explore the external factors about whether the different ECE environments or the length of clinical exposure has a stronger correlation with students’ learning achievement in the systems-based basic science (SBBS) course.

### Motivational beliefs and learning achievement

In addition to the ECE program as an attribute to high student achievement, individual differences among students, such as their attitudes towards learning, also affect their academic performance [18–21], and thus need to be taken into account. In the social cognitive perspective of motivation, psychologists, such as Atkinson and Pintrich, argued that individuals’ choices and persistence expended in performance can basically be predicted by expectations of achievement and the value attributed to the task (i.e., expectancy-value beliefs) [22–24]. There is considerable evidence supporting the direct connection between expectancy-value beliefs and academic achievement [25–31]. Expectation refers to students’ beliefs that they are capable of accomplishing a task, and self-efficacy is an essential component of expectation. Value (i.e., intrinsic task value) focuses on reasons why students engage in an academic task [32, 33]. In line with previous research on motivational beliefs [30, 34], this study focused on self-efficacy beliefs and intrinsic value (i.e., intrinsic task value), and their roles in students’ academic performance.

### Test anxiety in medical school

Whether test anxiety has a positive effect on medical students’ learning performance remains controversial. On the one hand, test anxiety might encourage learning and shift the students’ academic performance along the Yerkes-Dodson curve towards a more-optimal point. Medical students may be motivated by test-associated anxiety and stress [35–37]. On the other hand, test-associated stress and anxiety of medical students could cause their academic performance to deteriorate and impede their professional development [38].

### Gender

Findings on gender difference in medical students’ academic achievements have been very interesting and illuminating though findings have differed from one study to another. Alzahrani, Park, and Tekian for instance found that medical students’ study habits and methods differ by gender and have significant impact on performance outcomes of learners [39]. But the study by Al Shawwa et al. find no significant correlation between gender and academic achievements of medical students (*p* = 0.795) [40].

### Objectives

The aim of this study is to investigate correlations among students’ ECE experience, their motivation and test anxiety to the basic sciences, and their academic performance. Therefore, we have specified our research question as follows: Which factor has a stronger correlation with medical students’ academic performance in basic science: test-anxiety, motivational beliefs, or the ECE experience? Accordingly, competing hypotheses were raised as follows: Does the length of ECE affect students’ learning achievement in basic science and does the environment/setting where ECE takes place affect students’ learning achievement in basic science?; Do students’ motivational beliefs in learning basic science affect their achievement in basic science?; and Does test anxiety affect students’ learning achievement in basic science? In order to testify the hypotheses, the measurable objectives would be ECE experience (length and enviroment), attitudes toward basic medicine (motivational beliefs and test anxiety), gender, and learning performance in basic science. In short, this study would contribute to the field of medical education by investigating how does ECE experience, under the other factors such as test-anxiety and motivational beliefs, influence medical students’ learning performance in basic science.

## Methods

### Sample

**Fig. 1:**
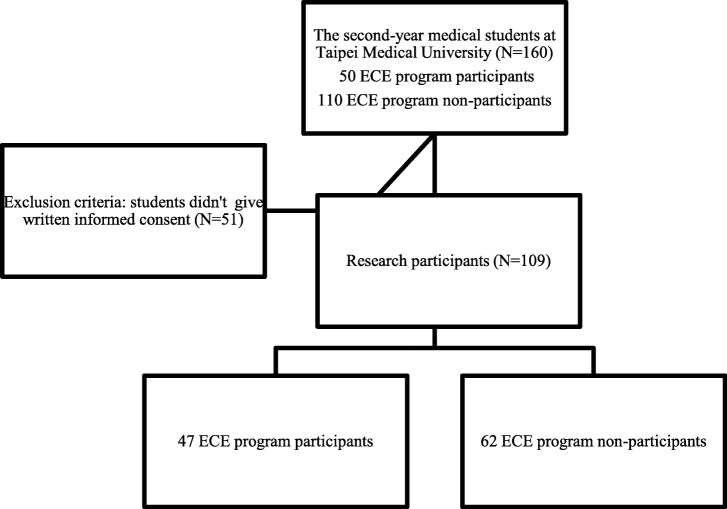
The Flow Chart of Research Participants Recruitment

This correlational study was conducted in the fall semester 2016 at Taipei Medical University. The target population of this study consists of all the second-year medical students who had early clinical exposure experiences. The subjects were selected from the School of Medicine, using convenience sampling and census method. A total of 160 sophomores were enrolled in this study. Fifty of them were the ECE course participants, while the others did not participate in the ECE program. However, only 109 of these 160 students gave written informed consent to participate in this study (47 ECE program participants and 62 non-ECE program participants). The process of the participants recruitment for this study is shown in the flow chart below (Fig. 1).

### Measures

#### Length of the ECE

The length of the ECE represents students’ involvement in the ECE program on the one hand, as they could decide how much time to spend doing ECE. Hence, each ECE program participant kept a record of his/her learning portfolio provided by the mentor. On the other hand, the length of ECE also denotes how long students participated in this kind of educational intervention. The learning portfolios of all ECE program participants were evaluated by the principal investigator of this study. The average length of ECE in group A amounted to 20.9 (range 9.5~26) hours, in group B to 22.5 (range 14~24.5) hours, and in group C to 23.06 (range 10~35.8) hours. In addition to the ECE course, all sophomores, whether participating in ECE or not, had at least 2 h of ECE experience, because each sophomore was required to attend a 2-h interview on relatives of body donators for their anatomy laboratory course. This interview activity tallied with the aforementioned definition of ECE, when medical students had contact with a former patient’s family.

#### Learning performance in the SBBS course

The SBBS course is the first subject matter for basic medicine that is given after the spring semester of the second-year curriculum according to the undergraduate medical doctor program at Taipei Medical University (TMU). The curriculum prior to the SBBS course is very similar to a liberal arts education. The SBBS course embraces the five following units: skeletal-muscular system, nervous system, pulmonary and circulatory systems, digestive system, and urogenital and endocrine systems. The midterm and final assessments of student performance in SBBS consists of 50 physiology related multiple-choice questions and 40 anatomy related fill-in the blank questions. Students’ test scores were collected at the end of the semester. In terms of the academic performance in the basic sciences, students’ overall test scores in the SBBS course were considered a variable in the multivariate linear regression analysis.

#### Students’ academic motivational beliefs and test anxiety scale

The authors generated a scale based on original scales created by Pintrich and used it in the study [22, 30]. This scale was used to measure students’ learning motivation in regard to the SBBS course. The questionnaire included areas of self-efficacy (eight items), intrinsic value (six items), and test anxiety (four items). For each questionnaire item, a 5-point Likert scale from 1 (strongly disagree) to 5 (strongly agree) was applied. One hundred nine research participants completed the questionnaire and provided written informed consent in March 2016, 1 week before their midterms. An item analysis for reliability and an exploratory factor analysis of the pretest results (*N* = 109) were conducted using SPSS 20 for Windows (SPSS, Chicago, IL, USA). The internal consistency reliabilities of the self-efficacy (Cronbach’s α = 0.813), intrinsic value (Cronbach’s α = 0.866), and test anxiety (Cronbach’s α = 0.866) were favorable.

### Ethical approval and research database

This study was approved by the Institutional Review Board of Taipei Medical University (N201609020). The authors confirm that the data supporting the findings of this study are available within the article (DOI: 10.6084/m9.figshare.5876823) and its Additional files 1, 2 and 3.

### Statistical analyses

Hierarchical multiple regression (HMR) analyses including three sequential regression models were performed to examine variable relationships. The first regression model (model I) included gender and the length of ECE as independent variables. The second regression model (model II) included motivational beliefs and test anxiety as additional independent variables. A measure of the ECE learning environment was added to the final regression model as three dummy variables (model III). The significance of the *F*-test indicated the goodness of the three multiple regression models, while the change in significance of the *F*-test helped us comparatively determine the best model. In addition, regression coefficients, an unstandardized β with its standardized β, were used to depict relationships between independent variables and students’ academic performance in basic science. The *R*^2^ statistic was reported for each regression model, reflecting the proportion of the variance in the dependent variable that was explained by the independent variables in each regression model [41]. All statistical analyses were carried out using SPSS vers. 17.0.

## Results

Table 2 presents results of the HMR analyses with three sequential regression models. As shown in model I, the regression model itself did not significantly pass the *F*-test (0.816 > 0.05), and neither gender nor the length of ECE had predictive power on students’ academic performance. Model II showed that the model itself had significant goodness, but also students’ test anxiety toward basic science had significant positive predictive power on their academic performance. In both models II and III, students’ self-efficacy beliefs and intrinsic value toward basic sciences had no significant influence on their academic performance. However, there were some findings worthy of note in model III, the best option among the three models. First, test anxiety identified in model II remained significantly influential on students’ academic performance in model III, which means test anxiety is a constant and crucial factor influencing SBBS. Second, the length of ECE became significantly predictive of students’ academic performance in model III, in contrast to its non-predictive power in models I and II. A noteworthy finding here is that when the ECE environment factors was considered and three dummy variables were included in model III, the length of ECE showed its significant yet negative effect as its standardized β(− 0.299) indicated, which seemingly implied medical students might spend their time on ECE at the price of academic performance. Third, beside the length of ECE, model III in advance showed the crucial effect of ECE environment: the variables of whether students were in ECE group B or C significantly and positively influenced their academic performance in basic sciences, while the variable of whether students were in ECE group A had no significant correlation with their academic performance. Being in group B or C respectively increased the basic science test score by 10.368 and 13.206 points, after controlling for other confounders in the model.

**Table 2:** Hierarchical multiple regression analysis on SBBS: gender, ECE-length, motivational beliefs, test-anxiety, and ECE-environment

Dependent: SBBS	Model I	Model II	Model III
	*B* (standardized)	*B* (standardized)	*B* (standardized)
Constant	69.373^***^	56.652^***^	52.425^***^
Gender	−0.376	1.080	1.209
Length of ECE (in hours)	−0.057 (− 0.058)	−0.028 (− 0.028)	−0.293 (− 0.299)^*^
Self-efficacy score		−0.469	− 0.282
Intrinsic value task score		0.490	0.323
Test anxiety score		1.184 (0.332) ^**^	1.094 (0.307) ^**^
Group A or not			5.497
Group B or not			10.368 (0.238)^*^
Group C or not			13.206 (0.337)^**^
*F*-sig (*p* value)	0.816	0.006^**^	0.002^**^
Change in *F*-sig (*p* value)	0.816	0.001^**^	0.035^*^
*R* ^2^	0.004	0.114	0.215
*N*	109	109	109

^*^p < 0.05; ^**^p < 0.01; ^***^p < 0.001

According to the *R*^2^ for each model as well as *F*-sig and changes in *F*-sig reported in Table 2, model III was better than the other two models. On the one hand, the *R*^2^ value indicated that model II explained 11.4% of the variance in students’ academic performance, an 11% increase over that (0.4%) of model I, and model III explained 21.5% of the variance, a 10.1% increase over that (11.4%) of model II. These two improvements in model comparison were significantly proven by changes in *F*-sig (0.006 < 0.01 and 0.002 < 0.01). On the other hand, we found that measures of students’ attitude toward learning basic science (self-efficacy, intrinsic value, and test-anxiety) in model II accounted for an additional 11% of the variance (the improvement in *R*^2^ from model I to model II is 0.114–0.004 = 0.11, 11%), and categorization of the ECE environment in model III explained another additional 10.1% of the variance in the dependent variable (the improvement in *R*^2^ from model II to model III is 0.215–0.114 = 0.101, 10.1%). In sum, adding new factors and variables increased the explanatory power.

## Discussion

According to the literature, the ECE experience, student’s motivational beliefs, and test anxiety may all be correlated with medical students’ academic performance in basic sciences [16, 20]. This study investigated which factor had the strongest correlation.

Alzahrani, Park, and Tekian [39] found that medical students’ study habits differ from gender, and study habits have signicant impact on their academic achievement. However, this study finds no significant correlation between gender and students’ academic performance.

Student’s motivational beliefs and test anxiety were considered as individual differences in learning [18]. Although a few studies found the effect of the preceptorship program on improving nursing students’ self-efficacy and learning outcomes [19], this study shows no significant correlation between students’ self-efficacy, their clinical exposure experiences, and their learning outcomes. One reason could be due to the different curricular i.e. core competency expectations among nursing and medical schools. Regarding individual differences, students’ test anxiety toward basic science had significant positive predictive power on their academic performance. This finding may be related to the processing efficiency theory proposed by Eysenck and Calvo, who argued that anxiety can cause an increment in on-task efforts and activities designed to improve performance [36].

Results of the HMR analyses showed that students who attended the ECE in certain hospitals (group B and C) had significantly better academic performance in the SBBS course. It should also be noted that the retention rate of course participants in group A was lower than the in groups B and C. The inconvenience of a long distance and long travel times did not seem to cause a low retention rate for students in groups B and C. On the contrary, more students in group B and C spent over 24 h in ECE. This finding might be explained by the aspect of culturalism, namely social learning takes place through participation in the community of practice. Each community of practice develops around mutual goals and interests over time [17]. Hence, we presumed that a variety of organizational cultures may exist among the three hospital groups. Considering the environmental factors of ECE, Widyandana, Majoor, and Scherpbier illustrated quality differences of preclinical training environments based on three types of setting: primary, secondary, and tertiary healthcare centers [42]. However, all three hospital groups in this study belonged to urban tertiary teaching hospitals.

We recognize some limitations within this study. For example, students’ prior academic ability was excluded because most of their previous compulsory courses were under the liberal arts curriculum. Also, it is unclear that whether students’ test achievement in previous subject matter could be a cognitive predictor for their learning achievement in the basic science. Another limitation is that the study did not did not measure organizational/−cultural differences or the mentor-mentee relationship among the different ECE environment as evaluation predictors of ECE outcomes. Therefore, questionnaires to investigate the pedagogical atmosphere during clinical exposure [43], students’ perception of social support [44], the organization of placement [45], and “patient mix” i.e., the diversity and quality of patient contacts [46], need to be adapted in future studies.

## Conclusions

In summary, this study concludes that the medical students with higher test-associated anxiety were able to perform better in their systems-based basic sciences test. The test-associated stress served as motivation for Medical students to perform well. Furthermore, there is a positive correlation between students’ learning achievement in basic medicine and their clinical exposure environment. Hence we suggest including further investigations of different ECE learning environments in future studies on ECE experiences.

## Additional files

Additional file 1: Raw data. (XLSX 21 kb)

Additional file 2: Data DOI. (TXT 163 bytes)

Additional file 3: Descriptive statistics. (CSV 423 bytes)

## Abbreviations

ECE: Early clinical exposure
HMR: Hierarchical multiple regression
SBBS: Systems-based basic sciences
TMU: Taipei Medical University
